# The Effects of Non-Steroidal Anti-Inflammatory Drugs Used for Orthodontic Pain Management on Tooth Movement: A Comprehensive Review of the Literature

**DOI:** 10.3390/jcm14092920

**Published:** 2025-04-23

**Authors:** Ioana-Maria Colceriu-Șimon, Dana Feștilă, Hanțig Emoke, Amelia Pancsur, Mara Ștefania Șimon, Cristian Doru Olteanu, Mihaela Păstrav, Olimpia Bunta, Mircea Ghergie

**Affiliations:** 1 Department of Orthodontics, Faculty of Dentistry, “Iuliu Hațieganu” University of Medicine and Pharmacy, 400012 Cluj-Napoca, Romania; simon.ioana@umfcluj.ro (I.-M.C.-Ș.); hantig.emoke@elearn.umfcluj.ro (H.E.); amelia.lavi.precup@elearn.umfcluj.ro (A.P.); olteanu.cristian@umfcluj.ro (C.D.O.); mihaela.pastrav@umfcluj.ro (M.P.); nemes.olimpia@umfcluj.ro (O.B.); mircea.ghergie@umfcluj.ro (M.G.); 2 Department of Dental Propaedeutics and Aesthetics, Faculty of Dental Medicine, “Iuliu Hațieganu” University of Medicine and Pharmacy, 400012 Cluj-Napoca, Romania; simon_mara_stefania@elearn.umfcluj.ro

**Keywords:** orthodontic pain, orthodontic tooth movement, non-steroidal anti-inflammatory drugs

## Abstract

Orthodontic treatment is commonly associated with pain, leading to reduced patient compliance and treatment adherence. Non-steroidal anti-inflammatory drugs (NSAIDs) are effective in reducing this pain by inhibiting prostaglandin synthesis. However, this mechanism may also interfere with orthodontic tooth movement (OTM) by affecting bone remodeling. This narrative review investigates the existing literature published between 2004 and 2024 to assess the impact of various NSAIDs on OTM and identify those that balance pain relief with minimal impact on tooth movement. Evidence shows that NSAIDs such as aspirin, ketorolac, diclofenac, and nimesulide significantly reduce OTM. The results for ibuprofen, meloxicam, and celecoxib were inconsistent with both no influence or a reduction in OTM, depending on dosage, mode, and duration of administration. Conversely, tenoxicam, nabumetone, etoricoxib, and parecoxib appear to have no effect on OTM. Among these, etoricoxib appears particularly promising due to its favorable gastrointestinal profile, high COX-2 selectivity, and negligible influence on OTM in clinical doses. However, the limited number of human trials highlights the need for further research to develop evidence-based guidelines for pain management that preserve treatment efficiency in orthodontics.

## 1. Introduction

Orthodontic pain is an unavoidable consequence and one of the most common side effects of orthodontic treatment. Numerous modalities, including pharmacological and non-pharmacological approaches, have been developed to alleviate orthodontic pain and discomfort in clinical practice [1,2]. The most commonly used pain management drugs for relieving orthodontic pain are acetaminophen (paracetamol) and non-steroidal anti-inflammatory drugs (NSAIDs) [3].

Acetaminophen is a widely used analgesic, but despite its structural similarity to NSAIDs, it lacks anti-inflammatory effects in peripheral tissues [4]. NSAIDs have been widely shown to be effective in managing orthodontic pain [5,6]. NSAIDs effectively reduce orthodontic pain by inhibiting cyclooxygenase (COX) enzymes, which block prostaglandins (PGs) involved in both pain and tooth movement. While this reduces inflammation, it may slow tooth movement by limiting prostaglandin E2 (PGE2)-driven bone remodeling. Traditional NSAIDs inhibit COX-1 and COX-2, offering pain relief but posing gastrointestinal risks, whereas selective COX-2 inhibitors (coxibs) reduce these risks but may increase cardiovascular concerns. Despite their impact on tooth movement, NSAIDs remain preferred for their anti-inflammatory benefits, while acetaminophen, which does not inhibit PG synthesis, is considered an alternative with a different, less effective mechanism against orthodontic inflammation. However, there remains an ongoing debate about the potential of NSAIDs to slow down the rate of tooth movement, and their use in the orthodontic field has been generally discouraged [7,8,9,10].

There is no clear scientific consensus on the optimal NSAIDs with minimal side effects for orthodontic treatment that ensure both clinical precision and patient well being. Considering this, our study reviews the existing literature on the relationship between tooth movement and orthodontic pain, evaluates the role of NSAIDs in pain and their impact on orthodontic tooth movement, and provides evidence-based recommendations to optimize pain relief while minimizing adverse effects on orthodontic treatment.

## 2. Materials and Methods

An electronic search of the literature was performed using PubMed and Google Scholar, as detailed in Table 1. This search employed specific keywords in English, which included “orthodontic pain”, “orthodontic tooth movement”, and “non-steroidal anti-inflammatory drugs”. Articles included in the review for the analysis of the effects of NSAIDs on orthodontic tooth movement (OTM) were original research articles (experimental or clinical) describing the effects of local or systemic administration of NSAIDs on OTM published between 2004 and 2024 with available full-text access in the English language. Studies had to assess the effects of NSAIDs on orthodontic tooth movement using objective methods such as histological, biochemical, or radiographic analysis. Studies were excluded if they were duplicates, editorials, opinions, correspondences, or reviews, were older than the included timeframe, had restricted access, lacked control groups, had small or undefined sample sizes, or provided insufficient methodological details. The initial database search yielded a number of 152 articles; however, the rigorous application of our inclusion and exclusion criteria refined this selection to a final set of 22 articles that met the eligibility requirements.

## 3. Results

### 3.1. Characteristics of Orthodontic Pain and Tooth Movement

The International Association for the Study of Pain defines pain as “an unpleasant sensory and emotional experience associated with actual or potential tissue damage, or described in terms of such damage” [11]. Orthodontic pain, specifically, refers to orofacial discomfort caused by OTM and is commonly characterized as soreness, pressure, and tension in the affected teeth [12,13].

Pain is a subjective experience influenced by factors such as age, gender, and psychological well being, which explains the variability in how patients perceive it [1]. Surveys of orthodontic patients indicate that pain is frequently reported as one of the most negative aspects of orthodontic treatment and a significant reason for considering discontinuation of care [13,14,15].

Orthodontic pain can occur at nearly every stage of treatment, including initial wire engagement, banding, wearing elastics, rapid maxillary expansion, braces removal, and separator placement [16]. Research indicates that orthodontic pain typically begins around 12 h after applying orthodontic forces, peaks within 24 h, gradually subsides over the next 3 to 7 days, and returns to baseline levels after approximately one month [12,16]. This pain can significantly impact patients’ quality of life by impairing chewing and speaking abilities, inducing emotional stress, and even leading to temporary challenges with learning and memory [17,18].

Orthodontic pain is mainly caused by an inflammatory reaction in the periodontium, which accompanies OTM. When force is applied to the crown of a tooth, it is transmitted to the periodontal ligament and alveolar bone. OTM is a process in which applying force induces bone resorption on the pressure side and bone apposition on the tension side [19,20,21]. Under normal conditions, the movement is highly coordinated, and the bone remodeling process is very efficient due to the coupling of bone resorption followed by bone formation. Alveolar bone adaptation to mechanical strains requires a minor, reversible injury to the periodontium as part of a physiological process [22].

When orthodontic forces are applied to teeth, a cascade of proinflammatory mediators is activated due to the compression of the periodontal ligament, leading to cellular, vascular, neural, and immunological reactions, which ultimately result in orthodontic pain and tooth movement [21]. Orthodontic pain and tooth movement are interrelated and dependent biological events, with local inflammation being their common mechanism [17].

When optimal forces are applied to teeth, the vascular vessels are compressed, and local ischemia develops [23]. Upon vascular compression and ischemia, anaerobic respiration is activated, causing local acidosis. The proton ion H^+^ binds to sensory endings and elicits painful sensations, which are transmitted to trigeminal neurons [12]. These painful sensations stimulate the release of several neurogenic mediators including, but not limited to, substance P, which is responsible for local vascular dilatation and local inflammation [24].

Substance P stimulates the production of RANK-L (receptor activator of nuclear factor-kappa ligand), which plays a critical role in OTM by regulating bone remodeling. It promotes the differentiation and activation of osteoclasts, the cells responsible for bone resorption. During tooth movement, increased RANK-L expression at the pressure sites facilitates bone resorption, allowing the tooth to shift into the desired position [22].

It is well known that the release of these neurogenic mediators stimulates the production of prostaglandins (PGs) in periodontal cells, enhancing inflammation and orthodontic pain, by binding to sensory endings in the periodontium [25]. Moreover, local acidosis and ischemia stimulate periodontal cells to release nitric oxide to increase vascular permeability [26]. Once vascular permeability increases, numerous leukocytes such as neutrophils, monocytes, and lymphocytes are recruited and release abundant inflammatory mediators, which further amplify local inflammation and bone remodeling due to their ability to stimulate osteoblast and osteoclast differentiation [27].

As orthodontic pain progresses, endogenous opioid molecules are activated to alleviate pain and prevent damage to periodontal tissue by promoting neovascularization and bone remodeling [28]. When orthodontic forces are reapplied, this process restarts [20,29].

### 3.2. Effects of NSAIDs on Orthodontic Pain and Tooth Movement

NSAIDs have been widely used for decades to alleviate orthodontic pain, and their analgesic, antipyretic, anti-inflammatory, and antiplatelet effects are well established [30]. These effects are primarily achieved through the inhibition of COX enzymes—COX-1 and COX-2—which are essential for the synthesis of prostaglandins (PGs). PGs are lipid mediators derived from arachidonic acid and belong to the eicosanoid hormone family. They are synthesized by two isoenzymes: COX-1, a constitutive enzyme present in most tissues and organs that maintains physiological homeostasis, and COX-2, an inducible enzyme expressed only in response to specific environmental stimuli [4,13,20]. PGs, particularly prostaglandin E2 (PGE2), play a significant role in orthodontic tooth movement by inducing pain via sensory nerve endings and promoting inflammation and bone remodeling [2,30]. PGE2 enhances vasodilation, increases vascular permeability, activates osteoclasts, and stimulates bone resorption—all processes that accelerate tooth movement [8,31]. By inhibiting the synthesis of PGE2, NSAIDs limit osteoclast recruitment and activation, thereby reducing bone resorption on the pressure side of the periodontium and ultimately slowing down OTM [32].

Additionally, NSAIDs interfere with the RANK/RANKL/OPG signaling pathway, which is essential for osteoclastogenesis. RANK (receptor activator of nuclear factor kappa-B) is a receptor found on the surface of osteoclast precursors, while RANK-L is its corresponding ligand expressed by osteoblasts and stromal cells. Their interaction promotes osteoclast differentiation and activation. OPG (osteoprotegerin) acts as a decoy receptor that binds to RANK-L, preventing it from interacting with RANK, thereby inhibiting osteoclastogenesis. The suppression of PG synthesis results in decreased expression of RANK-L, which reduces osteoclast differentiation [22].

NSAIDs also modulate neurogenic inflammation, another important mechanism in OTM. Substance P, a neuropeptide released in response to orthodontic forces, enhances inflammation and upregulates RANKL expression. By attenuating the release of substance P and other neuropeptides, NSAIDs further suppress osteoclast activity [24,25]. Moreover, NSAIDs may influence the expression of matrix metalloproteinases (MMPs), particularly MMP-13, which is involved in extracellular matrix degradation during bone remodeling. Research suggests that NSAID use can reduce MMP-13 expression, thereby limiting the periodontal tissue remodeling essential for efficient tooth movement [29].

There are different types of NSAIDs depending on how they influence the COX enzyme activity [4]. The main NSAIDs identified in the literature for orthodontic pain management are summarized in Table 2.

Non-selective COX inhibitors are the so-called “traditional NSAIDs”, and they inhibit both COX-1 and COX-2 isoenzymes. Traditional NSAIDs offer several advantages, such as analgesic and anti-inflammatory efficacy, improved function due to rapid pain relief, reduced side effects on the recognizing nervous system, and a wide variety of agents available on the market. Despite their value, their main disadvantages are related to gastrointestinal toxicity and their antiplatelet effect [33].

In order to overcome the unwanted side effects, especially at the gastric level, while maintaining the desired anti-inflammatory effects, preferential and selective COX-2 inhibitors, known as “coxibs”, have been developed [4,13]. These drugs specifically inhibit the activity of the COX-2 enzyme, which in turn blocks the synthesis of PGs that cause pain and inflammation. Unlike traditional NSAIDs, coxibs do not inhibit COX-1 activity, which is crucial for protecting the gastrointestinal tract and maintaining platelet function [34,35]. This clear advantage of coxibs at the gastric level is contrasted by a documented increase in cardiovascular risk, which seems to be dose and interval dependent. The negative influence of coxibs is thought to stem from a thrombophilic effect due to an imbalance of prothrombotic and antithrombotic factors. However, studies suggest that short-term use of recommended doses of NSAIDs, including coxibs, does not pose significant cardiovascular risks [35,36].

Several studies suggest that conventional NSAIDs may share the cardiovascular risks associated with coxibs. The MEDAL study compared the COX-2 inhibitor etoricoxib with the preferential COX-2 inhibitor diclofenac in patients with osteoarthritis and rheumatoid arthritis. It found similar risks of cardiovascular thrombosis for both drugs during long-term use. Coxibs also showed lower gastrointestinal risk while maintaining efficacy and safety comparable to conventional NSAIDs [35,37].

Inflammatory factors are crucial for tissue remodeling and tooth movement. However, many orthodontic patients take NSAIDs for pain treatment, which suppress COX enzymes and the production of PGs, decreasing tooth movement rates [22]. This is why acetaminophen has been commonly recommended by many studies to be the best drug for relieving pain associated with orthodontic treatment [5,38]. Acetaminophen has a different mechanism of action compared to NSAIDs and, therefore, cannot be classified as an NSAID. Whereas NSAIDs block COX-1 and/or COX-2, acetaminophen is thought to block a third isoform, COX-3, which is expressed only in the brain and the spinal cord, but its mechanism of action is still not completely understood [39]. As a consequence, acetaminophen has minimal effects on PG synthesis and, consequently, bone resorption associated with OTM [5,30]. However, pain caused by orthodontic treatment is due to peripheral inflammation, which is more effectively countered by the stronger anti-inflammatory effect of NSAIDs.

### 3.3. Types and Effects of NSAIDs

Several studies have investigated the effect of different types of NSAIDs on tooth movement as compared to control/placebo groups or to the administration of other analgesics.

**Figure 1:**
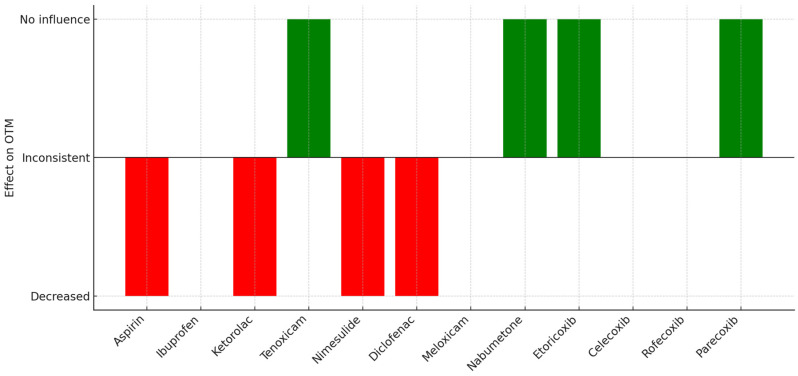
Summary of the effects of NSAIDs on OTM.

In the following section, the details of the included studies are summarized. Acetaminophen is not been described separately, as it is not a classical NSAID. The results of the database search, relevant to the topic of this review, are summarized in Table A1. A further summary of the effects of the discussed NSAIDs on OTM is provided in Table 3. To visually support the findings of this review, Figure 1 has been included to illustrate the differential effects of NSAIDs on OTM.

#### 3.3.1. Non-Selective COX Inhibitors

- Aspirin

Aspirin, also known as acetylsalicylic acid, is a powerful NSAID that is utilized to effectively reduce pain, fever, and inflammation, and it also functions as an antithrombotic. Aspirin acts by irreversibly modifying enzymes COX-1 and COX-2 [30,57].

Olteanu et al., in their study on rats, demonstrated a significant decrease in OTM in the groups in which aspirin and algocalmin were administered as compared to the control group without drug administration. Moreover, a statistically significant difference was identified when comparing OTM between the drug-administered groups, with the value being lowest in the group treated with aspirin. The histological study showed that in the control group, the alveolar bone displayed intense bone remodeling associated with orthodontic movement. However, in the group that received aspirin, no signs of bone remodeling were detected [40].

- Ibuprofen

Ibuprofen is a propionic acid derivative and was marketed in 1969 in the United Kingdom as Brufen. It was first used as an alternative to aspirin due to its greater tolerance [58]. The anti-inflammatory effect is achieved by blocking the synthesis of PGs in peripheral tissues [30].

Shetty et al., in their study on human subjects, analyzed the effect of ibuprofen and acetaminophen compared to the control group on PGE2 levels in the gingival crevicular fluid (GCF) during OTM. Quantitative evaluation of GCF samples collected from the subjects showed a statistically significant decrease in PGE2 levels in the experimental groups at 24 and 48 h compared to the control group. A highly significant difference in the mean concentrations of PGE2 was observed between the two experimental groups at both time points. This compelling evidence demonstrates that ibuprofen significantly suppresses PG synthesis compared to acetaminophen during the initial and subsequent days of OTM [32].

Arias and Marquez-Orosco compared, in their study, the effects of aspirin, ibuprofen, and acetaminophen on OTM and histologically evaluated the differences in bone resorption in the pressure area in rats treated with these analgesics. Similar to Olteanu et al.’s study, they concluded that NSAIDs, such as aspirin and ibuprofen, diminish the number of osteoclasts, probably by inhibiting the production of PGs, thereby reducing OTM [41].

However, Tuncer et al., in their double-blinded, randomized, placebo-controlled clinical study on the effects of ibuprofen and acetaminophen on PGE2 levels during OTM, showed that there were no statistically significant differences between the two analgesic groups regarding PGE2 levels. The authors concluded that OTM is a multifactorial process that cannot be regulated by only one chemical mediator. Short-term analgesic use during the most painful days of fixed appliance placement does not interfere with OTM. On the other hand, special attention should be given to patients with chronic illnesses, such as osteoarthritis, juvenile rheumatoid arthritis, or gout, where long-term analgesic treatment is required [43].

- Ketorolac

Ketorolac is an NSAID commonly used to manage moderate to severe pain, as well as conditions such as rheumatoid arthritis, osteoarthritis, ankylosing spondylitis, menstrual disorders, and headaches.

Rodríguez-Montaño et al. conducted a double-blinded, randomized clinical trial to compare the effects of ketorolac and acetaminophen on RANK-L expression in GCF during OTM. They concluded that both ketorolac and acetaminophen may reduce bone remodeling and potentially interfere with OTM. However, they emphasized the need for further studies with larger sample sizes to identify the most suitable analgesic that effectively manages pain without prolonging the duration of orthodontic treatment [44].

- Tenoxicam

Tenoxicam, an NSAID with analgesic and antipyretic properties, is used to treat osteoarthritis, backache, rheumatoid arthritis, and acute pain [59]. Arantes et al. studied the effect of the oral administration of tenoxicam on the OTM of maxillary canines in a randomized controlled double-blinded cross-over study compared to the control group. They concluded that tenoxicam did not influence the OTM of the upper canines. The authors selected tenoxicam as an NSAID because its long elimination half-life allows for once-daily dosing, providing effective control of mild to moderate acute pain, such as that caused by orthodontic activation, without notable adverse effects [60].

#### 3.3.2. Preferential COX-2 Inhibitors

- Nimesulide

Nimesulide is a mild inhibitor of PG synthesis and selectively targets COX-2. Its anti-inflammatory effect is achieved by reducing the production of superoxide by neutrophils and inhibiting the synthesis of platelet-activating factor [30]. It is used to treat short-term pain after dental surgeries, sports injuries, and primary dysmenorrhea [4,30].

A biochemical and histological study conducted by Tarvade et al. in guinea pigs showed that the administration of nimesulide and ibuprofen significantly decreased the rate of OTM and acid phosphatase levels in serum compared to the acetaminophen and control group. Moreover, the administration of nimesulide and ibuprofen significantly modified the appearance of osteoclasts in comparison to the control and acetaminophen group but was not significantly different between the two experimental groups. A high correlation was found between histological and biochemical findings. Thus, it can be concluded that the level of acid phosphatase in the serum reflects the turnover of alveolar bone during OTM [42].

- Diclofenac

Diclofenac is a monocarboxylic acid derived from acetic acid that inhibits PG synthesis by acting preferentially on the COX-2 isoenzyme. It has analgesic, antipyretic, and anti-inflammatory properties, and is marketed in the form of sodium and potassium salts for oral administration [61]. It is one of the most widely used NSAIDs, and it is commonly used in the treatment of rheumatoid arthritis, osteoarthritis, spondylitis, toothache, dysmenorrhea, and inflammatory conditions following trauma or surgery. Diclofenac provides rapid relief from pain and swelling [30].

In their study on rats, Knop et al. showed that the administration of potassium diclofenac inhibited bone resorption during the initial period of OTM, as evidenced by fewer blood vessels, Howship lacunae, and osteoclast-like cells histologically when compared to the control group. These findings indicate that potassium diclofenac suppresses bone resorption during the early stages of OTM [46].

- Meloxicam

Meloxicam selectively inhibits COX-2 enzyme, offering a more favorable side effect profile compared to both “traditional NSAIDs” and pure COX-2 inhibitors. Although recent clinical research has demonstrated that the analgesic efficacy of meloxicam administered prior to separator placement is comparable to that of acetaminophen and ibuprofen, there is currently limited information regarding its potential side effects on OTM [62].

In their study on rats, Kirschneck et al. used cone beam computed tomography to quantify OTM velocity after oral meloxicam administration. By inhibiting PG synthesis, meloxicam appears to downregulate inflammation and RANKL-induced osteoclastogenesis, resulting in a reduced OTM velocity of about 50%. This effect limits its suitability for use as analgesia during orthodontic therapy. However, the authors concluded that its good gastric tolerance profile suggests potential for future prophylactic use, which warrants further investigation [48].

- Nabumetone

Nabumetone is an effective NSAID drug that is rapidly converted in the liver into its active metabolite, 6-methoxy-2-naphthyl acetic acid. This active metabolite preferentially inhibits COX-2 activity and is responsible for the therapeutic effects of nabumetone [63].

Villa et al. observed in their study on humans the pulp-dentinal reactions, root resorption, tooth pain, and tooth movement after the application of a 4-ounce intrusive orthodontic force to human maxillary first premolars in patients given nabumetone. Their results showed that the use of nabumetone does not inhibit OTM when compared to the control group [49].

#### 3.3.3. Selective COX-2 Inhibitors (coxibs)

- Etoricoxib

Etoricoxib (Arcoxia) is currently the coxib with the highest COX-2 selectivity available and the only coxib specifically approved for the management of dental postoperative pain [50,64]. It has demonstrated excellent analgesic efficacy with significantly fewer side effects compared to traditional NSAIDs, as confirmed by multiple reviews [65,66]. Moreover, etoricoxib not only exerts minimal inhibitory effects on tooth movement and limited impact on the gastric mucosa and platelet function but it also acts as a potent and long-lasting pain reliever during orthodontic treatment, making it a potential alternative to acetaminophen [67]. A clinical trial by Gupta et al. that compared the effects of acetaminophen and etoricoxib to a placebo group confirmed that etoricoxib is significantly more effective in managing orthodontic pain than acetaminophen [13].

The experimental study conducted by Kirschneck et al. aimed to investigate the effects of different clinically relevant dosage regimens of etoricoxib on both OTM and cranial growth since the side effects of drugs are generally dose dependent. The study reported that OTM was significantly inhibited by about 33% only in rats receiving high doses of etoricoxib administered 7 days per week. In relation to its effects on orthodontic treatment, the researchers found that it had no significant impact on the rate of OTM at dosage regimens used in clinical practice to treat orthodontic pain [50].

A further study by Kirschneck et al. found that clinically relevant doses of etoricoxib had minimal impact on osteoclast activity, trabecular number, and bone remodeling during OTM in rats, with only slight inhibition observed at higher doses. They concluded that etoricoxib could be a viable alternative to acetaminophen as an analgesic in orthodontics [51].

Another clinical study conducted by Abdaljawwad et al. aimed to evaluate the effect of ibuprofen, acetaminophen, and etoricoxib on pain control and OTM in comparison to a placebo group. The results showed that all three drugs had no influence on the rate of OTM throughout the entire alignment and leveling period when used in recommended doses [7].

- Celecoxib

Celecoxib is a highly effective COX-2 inhibitor with low ulcerogenic potential, and it is used to treat mild to moderate pain due to its anti-inflammatory, analgesic, and antipyretic actions [68].

Hammad et al. studied the effects of different analgesics (celecoxib, ketorolac, and acetaminophen) on OTM and bone resorption compared to a control group using immunohistochemical staining of matrix metalloproteinase-13 (MMP-13) in rats. OTM requires significant remodeling of the periodontium, which is believed to be initiated in the periodontal ligament by MMPs. The number of MMP-13-positive osteoclasts was highest in the celecoxib-treated group, indicating that celecoxib administration did not reduce bone resorption or impair tooth movement in rats compared with other analgesics tested in this study [29].

Another study by Stabile et al. analyzed the effect of oral administration of acetaminophen and celecoxib on OTM in rats and found that treatment with both drugs when used for two days did not affect tooth movement. They concluded that short-term treatment with celecoxib may be a safe alternative medication for patients with acetaminophen hypersensitivity or hepatic disease [54]. Similarly, Jerome et al. concluded in their study on rats that the oral administration of celecoxib during the application of orthodontic forces does not interfere with OTM and may provide slight protection against root resorption [53].

A recent systematic review with a meta-analysis suggested that in the five included studies that analyzed the effect of acetaminophen, aspirin, and celecoxib in rats, the short-term (less than one week) use of celecoxib for relieving orthodontic pain might not inhibit OTM [2].

On the other hand, Gameiro et al., in their experimental study on rats, rejected the hypothesis that celecoxib administration had no effect on OTM. Although celecoxib did not interfere with the number of osteoclasts, their activity might be reduced, supporting the conclusion that both short- and long-term administration of celecoxib can inhibit OTM [55]. Furthermore, another study by Sodagar et al. showed that celecoxib injections significantly decreased OTM and osteoclast count in rats compared to the control groups. They suggested that this may result from COX-2 enzyme inhibition and the subsequent reduction in PG production [56].

Gonzales et al. compared, in their study on rats, the effect of oral administration of high and low doses of aspirin, acetaminophen, meloxicam, celecoxib, and prednisolone to that of a control group. Their results showed that only celecoxib significantly suppressed OTM, while aspirin, acetaminophen, and meloxicam did not seem to interfere with it [39].

- Other coxibs

A clinical study compared the effects of two different NSAIDs, aspirin and rofecoxib, on GCF volume and on PGE2 levels of the GCF during OTM in human subjects compared to a control group. Rofecoxib was not found to affect PGE2 levels significantly during the experimental period, but aspirin significantly inhibited PGE2 synthesis on the first day of the experiment. These results suggest that rofecoxib can be used as an analgesic to control pain without affecting the outcome of orthodontic treatment, though the authors concluded that further studies are recommended [31].

On the other hand, de Carlos et al. showed, in their study on rats, that rofecoxib and diclofenac both significantly inhibited OTM—partially in the case of rofecoxib and completely in the case of diclofenac. Nevertheless, no statistically significant difference was found between the effects of rofecoxib and diclofenac [47]. A further study by de Carlos et al. compared the effect of injectable administration of rofecoxib, celecoxib, and parecoxib on OTM in rats. Their results showed that rofecoxib completely inhibited OTM in rats, whereas celecoxib and parecoxib did not [52].

However, rofecoxib was withdrawn in 2004 from the U.S. and European markets by its manufacturer due to reports of increased cardiovascular events and skin rashes, respectively [6].

## 4. Discussion

This literature review examines various NSAIDs used in managing orthodontic pain during tooth movement. With the growing array of available medications, it is essential for orthodontists to stay informed, particularly about the mechanisms underlying each drug therapy and the clinical management of inflammatory symptoms customized to each patient. Moreover, patients may use NSAIDs for other medical conditions or independently of their orthodontic care, highlighting the need for orthodontists to be informed about their potential effects on the biomolecular pathways involved in tooth movement.

Traditional pain management methods rely on the administration of acetaminophen and NSAIDs. Acetaminophen is one of the most widely used analgesics for pain relief. However, its effects at the pain site are relatively weak and insufficient to provide substantial relief. While it has been shown to have no effect on tooth movement, similar to other non-opioid analgesics, acetaminophen exhibits a “ceiling effect,” where increasing the dose beyond a certain threshold does not enhance pain relief.

NSAIDs, in contrast, achieve analgesia primarily through the peripheral inhibition of PG synthesis, a key contributor to OTM-associated pain. However, NSAIDs may also reduce the rate of tooth movement. Concerns regarding the side effects of conventional NSAIDs have prompted the development of selective COX-2 inhibitors to reduce gastrointestinal toxicity.

Our study found that currently, only a limited number of human studies have examined the effects of NSAIDs on OTM. Most of these studies have been underpowered, lacked proper control groups, or provided low levels of evidence. Therefore, experimental studies were also reviewed, and the results were pooled to better understand the relationship between several NSAIDs and OTM (Table A1 and Table 3).

NSAIDs differ significantly in their impact on OTM (Figure 1), depending on factors such as COX selectivity, dosage, mode of administration, and duration of use. Differences in study outcomes likely reflect variations in experimental design—such as subject type (human vs. animal), drug delivery method, and how OTM is measured (Table A1). Standardizing factors, like appliance type and dosage, is essential for consistent results. Additionally, biological differences between species complicate generalization, highlighting the need for well-designed clinical trials. The differences in the included studies likely stem from methodological heterogeneity but could also appear because of differences in age, gender, and genetic background of the subjects. Most animal studies lacked demographic reporting, while human trials rarely included detailed age or gender data. These limitations hinder a comprehensive understanding of individual variability in response to NSAID treatment. Future research should include patient demographics and consider genetic factors—such as polymorphisms in COX, cytokines, and bone-regulating genes like RANK, RANK-L, and OPG—which may influence drug efficacy and OTM outcomes.

Clinical studies have shown that aspirin [31] and ketorolac [44] reduce OTM, ibuprofen may [32] or may not [7,43] affect OTM, while tenoxicam [60], nabumetone [49], etoricoxib [7], and rofecoxib [31] appear to have no significant influence on OTM (Table A1 and Table 3). A clinical comparison between aspirin and rofecoxib revealed that while aspirin significantly inhibited PGE_2_ synthesis, rofecoxib did not alter its levels, suggesting a minimal effect on OTM [31]. In a randomized clinical study, ketorolac was shown to decrease RANK-L expression relative to acetaminophen, indicating a potential reduction in bone resorption [44]. One study demonstrated that administering ibuprofen (400 mg) during appliance activation led to a significant reduction in gingival crevicular fluid PGE_2_ levels compared to acetaminophen (500 mg), implying an inhibitory effect on bone remodeling [32]. However, another trial with a similar design found no significant differences in PGE_2_ levels between the ibuprofen and acetaminophen groups over a seven-day period, indicating that short-term use may not significantly impact OTM [43]. Clinical evidence indicates that tenoxicam effectively manages orthodontic pain without significantly affecting tooth movement [60]. Nabumetone was observed to have minimal impact on tooth movement, with only a slight monthly decrease in displacement [49]. Finally, a study comparing acetaminophen, ibuprofen, and etoricoxib during the alignment phase found no significant differences in mesial tooth displacement when these drugs were used at recommended doses [7].

Due to the limited number of robust human studies, animal models have been crucial for assessing NSAIDs’ effects on OTM. Findings consistently show that NSAIDs can impair OTM by inhibiting prostaglandin synthesis and reducing osteoclast activity, with effects varying by drug type, dose, and administration. Non-selective NSAIDs such as aspirin [40,41], ibuprofen [41,42], and ketorolac [29] have shown marked reductions in tooth movement. Preferential COX-2 inhibitors, including diclofenac [46,47] and nimesulide [42], also significantly decrease movement. Among selective COX-2 inhibitors, experimental findings indicate that high doses of meloxicam [48] and rofecoxib [47,52] tend to inhibit tooth movement, whereas etoricoxib [50,51] and parecoxib [52] administered at clinically relevant doses exhibit minimal impact. Experimental studies on celecoxib have shown conflicting results regarding its influence on OTM, with either no effect or a slight reduction in OTM, depending on dosage, administration route, and duration. Several studies have found that short-term use of celecoxib does not significantly affect OTM. Hammad et al. [29], Stabile et al. [54], Jerome et al. [53], and De Carlos et al. [52] all reported no inhibitory effect on OTM, even with a local injection. A meta-analysis by Fang et al. supported these findings, concluding that short-term celecoxib use (<1 week) does not impair OTM [2]. In contrast, other studies have shown that celecoxib may reduce OTM. Gameiro et al. observed that both short- and long-term use significantly decreased OTM, possibly by suppressing osteoclast activity [55]. Sodagar et al. reported reduced OTM and osteoclast count following celecoxib injections [56]. Gonzales et al. found that celecoxib, unlike other analgesics tested, significantly inhibited OTM at both low and high doses [39]. The variation in outcomes across celecoxib studies can be attributed to several experimental factors. Variations are attributed to dose, duration, administration route (with local injections more inhibitory), study design, and species differences, as all studies were in rats. Biologically, celecoxib may suppress osteoclast activity without altering cell numbers, explaining the inconsistent findings. Given celecoxib’s favorable gastric safety and low ulcer risk, it may be a good option for short-term orthodontic pain in patients intolerant to acetaminophen. However, more controlled human trials are needed due to conflicting evidence.

Both clinical and experimental studies showed that etoricoxib emerges as a potentially favorable analgesic for orthodontic pain management. It may serve as an alternative to acetaminophen, which, while centrally acting on COX-3, does not impact OTM [39]. Additionally, etoricoxib’s longer half-life, requiring only once-daily administration, enhances patient compliance [13]. Its favorable safety profile reduces risks of allergic reactions, rhinitis, asthma, and liver damage often associated with high doses of acetaminophen. The molecular mechanism explaining why etoricoxib does not have a significant effect on OTM may be due to its minimal impact on osteoclast activity and RANK-L expression, key factors in bone resorption during OTM. Unlike non-selective NSAIDs, which inhibit both COX-1 and COX-2 and may strongly suppress bone remodeling, etoricoxib is the most COX-2 selective coxib currently available (COX-2/1 ratio of 344:1) that appears to allow for adequate PG activity at the local level, preserving normal OTM while still providing pain relief.

While etoricoxib has shown promise in managing pain, its role in orthodontic pain relief remains underexplored. Future research should focus on well-designed clinical studies to evaluate its efficacy, optimal dosage, and safety profile compared to traditional NSAIDs used in orthodontics. Additionally, comparative studies assessing different NSAIDs in orthodontic pain management are necessary to establish the most effective and well-tolerated option. Such investigations will help refine clinical guidelines and improve patient care by providing evidence-based recommendations for pain management in orthodontic treatments.

A chief limitation of this review is the lack of sound human clinical trials directly examining how specific NSAIDs influence orthodontic tooth movement. Many of the included studies were either animal based or small-scale clinical investigations, which restricts the ability to generalize findings and establish definitive protocols. Furthermore, the reviewed studies employed diverse methodologies—varying doses, administration routes, and types of orthodontic movements—making it challenging to compare outcomes in a standardized manner. These factors collectively limit the strength of the evidence and underscore the need for larger, well-controlled human trials. Despite these constraints, the review offers a comprehensive synthesis of data spanning both experimental and clinical research from 2004 to 2024, encompassing multiple classes of NSAIDs—non-selective, preferential COX-2 inhibitors, and selective COX-2 inhibitors. This wide range of studies helps clinicians and researchers understand how different NSAIDs affect orthodontic pain relief and tooth movement. Comparing multiple NSAIDs directly reveals subtle differences in their impact on bone remodeling, offering a broader view than studies focusing on just one drug.

## 5. Conclusions

Effective pain management is essential for patient compliance and comfort during orthodontic treatment. However, the use of NSAIDs must be carefully considered due to their potential to interfere with orthodontic tooth movement by altering bone remodeling processes. Evidence from both clinical and experimental studies shows that certain NSAIDs—such as aspirin, ketorolac, diclofenac, and nimesulide—can significantly reduce the rate of tooth movement, while others like tenoxicam, nabumetone, etoricoxib, and parecoxib appear to have minimal impact. The effects of ibuprofen, meloxicam, and celecoxib were inconsistent, influenced by factors such as dosage, route, and duration of administration. Among selective COX-2 inhibitors, etoricoxib appears particularly promising as a safe and effective analgesic with limited influence on OTM when used at clinically recommended doses. Due to the limited number of high-quality human trials, further research is necessary to establish clear, evidence-based guidelines for the use of NSAIDs in orthodontics. Until then, clinicians should balance analgesic efficacy with the biological implications of each drug to ensure optimal treatment outcomes.

## Figures and Tables

**Table 1:** Search strategy summary.

Items	Details
Databases searched	PubMed, Google Scholar
Search terms used	“orthodontic pain”, “orthodontic tooth movement”, “non-steroidal anti-inflammatory drugs”
Timeframe	2004–2024
Inclusion criteria	clinical and experimental studies, local or systemic administration of NSAIDs, objective methods of OTM evaluation, full-text articles, English language only
Exclusion criteria	duplicates, editorials, opinions, correspondences, reviews, older than the included timeframe, full text unavailable, articles not in the English language, lack of a control group, small or undefined sample sizes, insufficient methodological details

**Table 2:** Examples of different types of NSAIDs used for orthodontic pain management.

Types of NSAIDs	Examples
Non-selective COX-inhibitors	Aspirin, Ibuprofen, Ketorolac, Tenoxicam
Preferential COX-2 inhibitors	Nimesulide, Diclofenac, Meloxicam, Nabumetone
Selective COX-2 inhibitors	Etoricoxib, Celecoxib, Parecoxib, Rofecoxib

**Table 3:** Type of included studies and the effects of the discussed non-steroidal anti-inflammatory drugs on orthodontic tooth movement.

NSAIDs	Type of Study	Effect on OTM
Aspirin	Clinical [31]	Decreased
	Experimental (rats) [40,41]	Decreased
	Experimental (rats) [39]	No influence
Ibuprofen	Clinical [32]	Decreased
	Experimental (rats) [41]	Decreased
	Experimental (guinea pigs) [42]	Decreased
	Clinical [7,43]	No influence
Ketorolac	Experimental (rats) [29]	Decreased
	Clinical [44]	Decreased
Tenoxicam	Clinical [45]	No influence
Nimesulide	Experimental (guinea pigs) [42]	Decreased
Diclofenac	Experimental (rats) [46,47]	Decreased
Meloxicam	Experimental (rats) [48]	Decreased
	Experimental (rats) [39]	No influence
Nabumetone	Clinical [49]	No influence
Etoricoxib	Clinical [7]	No influence
	Experimental (rats) [50,51]	No influence
Celecoxib	Experimental (rats) [29,52,53,54]	No influence
	Experimental (rats) [39,55,56]	Decreased
Rofecoxib	Clinical [31]	No influence
	Experimental (rats) [47,52]	Decreased
Parecoxib	Experimental (rats) [52]	No influence

## Data Availability

Data sharing is not applicable. No new data were created or analyzed in this study. Data sharing is not applicable to this article.

## Abbreviations

The following abbreviations are used in this manuscript:

COX	cyclooxygenase
GCF	gingival crevicular fluid
MMP-13	matrix metalloproteinase-13
NSAID	non-steroidal anti-inflammatory drug
NSAIDs	non-steroidal anti-inflammatory drugs
OPG	osteoprotegerin
OTM	orthodontic tooth movement
PGs	prostaglandins
PGE2	prostaglandin E2
RANK-L	receptor activator of nuclear factor-kappa ligand

## Appendix A

The following appendix contains supplementary data and detailed results from the selected studies, which complement the main findings presented in the article. These materials are included for transparency and to facilitate further analysis.

**Table A1:** The results of the database search.

Authors, Year	Study Group, Sample Size, and Distribution	Substance Investigated	Applied Force, Movement	Administration Path, Frequency of Administration, Dosage	Study Duration	Method of Evaluation of OTM	Outcomes	Conclusions
1. Olteanu et al. [40], 2015	Wistar rats, 24, divided into 3 groups of 8 subjects each	Aspirin Algocalmin	25 g from a nickel–titanium closed-coil spring between the inferior first molar and left inferior incisor	Gastric gavage Every 2 days for 10 days, the next day after device application CG (Group I): no intervention EG1 (Group II): 1.5 mL aspirin (concentration 20 mg/mL) EG2 (Group III): 1.2 mL algocalmin (concentration 5 mg/mL)	28 days	Histological study for size of bone areola determination (μm) Distance from the initial position to the final position of M1 (mm)	Reduced size of the bone areola in EG1 (74) and EG2 (127) compared to CG (244) Reduced OTM in EG1 (0.03) and EG2 (0.19 ± 0.08) compared to the CG (3.61 ± 0.29)	OTM and bone remodeling were reduced more in the aspirin group
2. Shetty et al. [32], 2013	Humans, 42, randomly divided into 3 groups of 14 subjects each	Ibuprofen Acetaminophen	150 g from a nickel–titanium tension spring between the maxillary molars and canines, after extraction of premolars	Oral administration At the appliance activation for 2 days, 3 times daily CG: no intervention EG1: ibuprofen 400 mg EG2: acetaminophen 500 mg	7 days	Quantitative PGE2 levels in GCF samples from the maxillary canines using ELISA before (T0) and after spring activation at 24 (T1), 48 (T2), and 168 h (T3)	A statistically significant decrease in PGE2 levels in the EG1 at T1 (*p* = 0.002) and T2 (*p* = 0.011) when compared to CG. A statistically significant difference in the mean concentrations of PGE2 between the two EGs at T1 (*p* = 0.006) and T2 (*p* = 0.011)	OTM was reduced more in the ibuprofen group due to the inhibition of PGE2 synthesis
3. Arias and Marquez-Orosco [41], 2006	Wistar rats, 36, divided into 4 groups of 9 each	Aspirin Ibuprofen Acetaminophen	35 g from a 3-spin loop made of 0.016 in a beta-titanium alloy wire between the incisors	Gastric gavage Every 12 h for 10 days, diluted in 0.6 mL of reverse osmosis-filtered water CG: 0.6 mL of reverse osmosis-filtered water EG1: 100 mg/kg aspirin 500 mg EG2: 30 mg/kg ibuprofen 400 mg EG3: 200 mg/kg acetaminophen 500 mg	10 days	Histological analysis of the bone Average tooth movement of the incisors (mm)	Reduced numbers of resorption lacunae/osteoclasts during OTM in EG1 (1.86 ± 1.15/1.83 ± 1.18) and EG2 (2.00 ± 1.61/2.48 ± 2.25) compared with CG (6.09 ± 1.61/14.02 ± 5.27) and EG3 (5.86 ± 1.52/13.43 ± 4.31) (*p* < 0.01) Reduced tooth movement for EG1 (1.32 ± 0.28) and EG2 (1.22 ± 0.29) compared to CG (1.86 ± 0.53) and EG3 (1.80 ± 0.41)	OTM and the numbers of resorption lacunae and osteoclasts were reduced more in the ibuprofen and aspirin group
4. Tuncer et al. [43], 2014	Humans, 48, randomly divided into 3 groups CG—16 subjects EG1—17 subjects EG2—15 subjects	Ibuprofen Acetaminophen	0.014-inch archwire, non-extraction	Oral administration Two tablets, 1 h before the appointment and 6 h after bonding CG (group C): lactose placebo capsule EG1 (group A): ibuprofen 400 mg EG2 (group B): acetaminophen 500 mg	7 days	Quantitative PGE2 levels (pg/μL) in GCF samples from the maxillary canines with ELISA prior to bonding (T0), right after the bonding (T1), and on the first (T2), second (T3), third (T4), and seventh day (T5) after bonding	The PGE2 levels in the CG/EG1/EG2 were T0: 22.33 ± 17.21/14.53 ± 13.27/16.14 ± 12.59 T1: 16.81 ± 11.69/9.27 ± 4.81/10.89 ± 10.53 T2: 17.33 ± 13.53/19.30 ± 17.25/16.66 ± 14.39 T3: 21.37 ± 24.33/14.52 ± 13.78/21.78 ± 40.11 T4: 17.66 ± 14.90/9.96 ± 7.16/14.63 ± 11.35 T5: 16.18 ± 10.08/11.59 ± 11.84/15.91 ± 13.705	OTM was not influenced by 1–2 days of ibuprofen use as no time-related differences in the PGE2 level were found between the groups
5. Rodríguez-Montaño et al. [44], 2024	Humans, 24, randomly divided into 3 groups of 8 subjects each	Ketorolac Acetaminophen	Elastic separator between the upper molar and premolar	Oral administration One capsule every 8 h for 5 days CG (Group 1): placebo calcined magnesia 500 mg EG1 (Group 2): ketorolac 10 mg EG2 (Group 3): acetaminophen 500 mg	5 days	RANK-L concentrations (pg/µL) from GCF of the right upper first molar mesial zone with ELISA analysis at four time points: before pharmacological intervention (T0), at 24 h (T1), at 48 h (T2), on the 5th day (T3)	Increased RANK-L concentration at T1 in CG (0.146 ± 0.278) compared to EG1 (0.036 ± 0.021) and EG2 (0.047 ± 0.052). RANK-L concentrations at T2 in the 3 study groups did not show a significant difference (CG:0.033, EG1:0.032, EG2:0.033) At T3, RANK-L levels in the EG1(0.188 ± 0.446) group remained lower than in the CG (0.111 ± 0.118) and EG2 (0.041 ± 0.023) groups	OTM may be influenced by ketorolac through a decrease in RANK-L expression
6. Arantes et al. [60], 2009	Humans, 36, randomly divided into 3 groups of 12 subjects	Tenoxicam	Bilateral retraction of the upper canine teeth after premolar extraction with a nickel–titanium spring. Each retraction procedure consisted of three activations that were started on the right side and then alternated between the right and left sides at 14-day intervals	Oral administration, 45 min before orthodontic activation, after activation, 24 h, and 48 h after activation CG: placebo tablets at all time points EG1 (Group A): 20 mg tenoxicam + placebo + 20 mg tenoxicam at 24 and 48 h EG2 (Group B): placebo + 20 mg tenoxicam after activation, at 24 and 48 h. The rescue analgesic offered to the patients in all 3 groups was paracetamol, at a dose of 750 mg, up to four times a day	4 weeks	Measuring the distance between the canine and second premolar teeth with a caliper (mm), prior to activation and 4 weeks later	The orthodontic movement was statistically similar between CG, EG1, and EG2 4 weeks after each orthodontic activation (the distance between the canine and second premolar was between 0.8 and 1 mm in all study groups)	OTM was not influenced by tenoxicam administration
7. Tarvade et al. [42], 2013	Guinea pigs, 28, Group I, 24 for biochemical study Group II, 4 animals for histological study. Group I and Group II were further divided into 4 subgroups	Acetaminophen Ibuprofen Nimesulide	A 0.014” spring with two vertical loops between the mandibular central incisors	Oral administration, 12 hourly for 3 days CG (Subgroups I and II (a)), no drug administration EG1 (Subgroups I and II (b)), acetaminophen suspension EG2 (Subgroups I and II (c)), ibuprofen suspension EG3 (Subgroups I and II (d)), nimesulide suspension	3 days	The tooth separation measurements were performed between the mesial margins of the incisal edges of the two mandibular incisors using a Vernier caliper prior (mm) to placement (T0) and 24 h (T1), 48 h (T2), and 72 h (T3) after orthodontic appliance placement Acid phosphatase levels from blood samples 72 h after orthodontic appliance placement Histological study of the bone	At T3, the mean tooth separation was found to be highest in CG (3.70 ± 0.08), while minimal tooth separation was observed in EG2 (2 ± 0.08) and EG3 (1.75 ± 0.148)	OTM, acid phosphatase levels in serum, and the rate of bone resorption and appearance of osteoclasts were decreased by ibuprofen and nimesulide administration
8. Knop et al. [46], 2012	Wistar rats, 90, randomly divided into 3 groups of 30 each	Postassium diclofenac Dissodium phosphate	30 g force from a nickel–titanium closed-coil spring between the maxillary right first molar and maxillary central incisors. Animals were sacrificed 3, 7, or 14 days after placement of the orthodontic appliance	Intramuscular, daily CG (control): 0.9% saline solution EG1: 5 mg/kg postassium diclofenac EG2: 2 mg/kg dexamethasone dissodium phosphate	14 days	Histological analysis of the bone at the upper first molars by quantifying osteoclast-like cells, active Howship lacunae, and blood vessels and evaluation of bone neoformation	Reduced numbers of osteoclast-like cells, Howship lacunae, and blood vessels throughout all periods studied in the EG1 group compared to CG. Osteoclast-like cells: day 3 (EG1: 1.9 ± 0.74, CG: 5.8 ± 1.55), day 7 (EG1: 7.5 ± 2.95, CG: 16.9 ± 3.35), day 14 (EG1: 3.1 ± 1.45, CG: 3.3 ± 1.06). Howship lacunae: day 3 (EG1: 3.2 ± 1.03, CG: 6.4 ± 1.98), day 7 (EG1: 5.8 ± 3.73, CG: 17.8 ± 2.57), day 14 (EG1: 5.3 ± 1.95, CG: 3.9 ± 1.98). Blood vessels: day 3 (EG1: 14.7 ± 2.58, CG: 25 ± 3.02), day 7 (EG1: 16.8 ± 3.01, CG: 7.1 ± 1.45), day 14 (EG1: 14.7 ± 3.4, CG: 3.1 ± 1.98). At all time points, EG1 presented lower mature collagen deposition than CG: day 3 (EG1: 5.5 ± 2.7, CG: 10.78 ± 3.73), day 7 (EG1: 29.8 ± 8.13, CG: 39.55 ± 4.27), day 14 (EG1: 96.9 ± 2.08, CG: 100 ± 0)	OTM movement is reduced by potassium diclofenac as it inhibits bone resorption during the initial period of OTM and, consequently, there is a delay in collagen maturation during bone neoformation
9. Kirschneck et al. [48], 2017	Fischer-344 rats, 63, randomly divided into three consecutive experiments of 21 animals (A/B/C) in three experimental groups of 7 animals each. Experiment A quantified tooth movement velocity	Meloxicam	25 g from a modified nickel–titanium closed-coil tension spring between the molars and incisors	Oral gavage, 10 days prior to orthodontic force CG: no intervention EG1: orthodontic force EG2: orthodontic force with a daily oral 3 mg/kg meloxicam	28 days	Quantification of tooth movement velocity after 14 and 28 days of OTM by means of cone beam computed tomography	A significantly reduced mean tooth movement velocity was observed both within 14 and 28 days of OTM of M1 (day 14–64%, day 28–46%; *p* < 0.001) and for the mesialization of M2 (day 14–51%, day 28–54%; *p* < 0.001) in EG2. A significant reduction in the mesial drift of the third upper left molar in an anterior direction was also observed following meloxicam medication (day 14–40%, day 28–35%)	Meloxicam reduces PG synthesis that subsequently causes a corresponding reduction in the RANKL/OPG expression ratio and associated osteoclastogenesis, thus retarding OTM by about 50%
10. Villa et al. [49], 2005	Humans, 25, CG, 16 premolars, EG, 34 premolars	Nabumetone	113 g intrusive force on the first premolars from a 0.017 × 0.025 stainless steel archwire	Oral administration, 2 days before the orthodontic activation and for 4 more days after CG: two tablets placebo every 24 h EG: two tablets of nabumetone 500 mg every 24 h	8 weeks	Measurements with a digital Vernier calibrator (mm) on the initial casts of each patient and on casts taken after the orthodontic movement was made	Intrusive movement was CG: 1.711 and EG: 1.449 mm, with *p* = 0.02	The use of nabumetone does not block OTM. There was a decrease of only 0.13 mm per month
11. Kirschneck et al. [50], 2018	Fischer-344 rats, 40, randomly divided into 4 groups of 10 each	Etoricoxib	25 g from a modified nickel–titanium closed-coil tension spring between the first upper left molar and the upper ipsilateral incisor	Oral gavage One week prior to the start of OTM and continued daily until day 28 of OTM CG: 1.5 ml tap water per day for 5 weeks EG1: normal dose (7.8 mg/kg) etoricoxib, 3 consecutive days/week EG2: normal dose (7.8 mg/kg) etoricoxib, 7 days/week EG3: high dose (13.1 mg/kg) etoricoxib, 7 days/week	35 days	CBCT imaging at the orthodontic left jaw side at the start and end of the experiment	Anterior metric tipping of M1 was significantly inhibited (*p* = 0.046) by about 33% in EG3 (median = 0.5 mm) only compared to CG (median = 0.8 ± 0.2 mm), with a respective but insignificant tendency also detectable for the normal dosages	OTM is not influenced by clinically relevant dosage regimens of etoricoxib used in clinical practice to treat dental or orthodontic pain
12. Kirschneck et al. [51], 2020	Fischer-344 rats, 40, randomly divided into 4 groups of 10 each	Etoricoxib	25 g from a modified nickel–titanium closed-coil tension spring between the first upper left molar and the upper ipsilateral incisor	Oral gavage One week prior to the start of OTM and continued daily until day 28 of OTM CG: 1.5 ml tap water per day for 5 weeks EG1: normal dose (7.8 mg/kg) etoricoxib, 3 consecutive days/week EG2: normal dose (7.8 mg/kg) etoricoxib, 7 days/week EG3: high dose (13.1 mg/kg) etoricoxib, 7 days/week	35 days	OTM-associated dental root resorptions, osteoclastogenesis, trabecular number, and periodontal bone loss were quantified by histomorphometrical, histochemical, and microCT analyses of the dissected tooth-bearing upper jaw sections	Reduced trabecular number in CG (*p* = 0.0849) and EG1 (*p* = 0.0609), whereas in EG2 (*p* = 0.2449) and EG3 (*p* = 0.5786), this the effect was not present. Osteoclastogenesis and osteoclast activity were not significantly increased in any of the groups	Etoricoxib in clinically relevant doses does not affect osteoclastogenesis, trabecular number in the alveolar bone, and remodeling associated with OTM. Only a slight inhibitory effect on bone remodeling is to be expected at high dosages
13. Abdaljawwad and Al-Groosh [7], 2022	Humans, 40, randomly divided into 4 groups of 10 each	Acetaminophen Ibuprofen Etoricoxib	0.012-inch archwire was placed for alignment as a starting archwire, and the usual wire sequence was followed (0.014-inch, 0.016-inch, 18-inch NiTi) at 6-week visit intervals	Oral administration, 1 h before bonding and archwire placement and continued for 3 days including the bonding day CG: starch capsules once daily EG1: acetaminophen 500 mg thrice daily EG2: ibuprofen 400 mg thrice daily EG3: etoricoxib 60 mg once daily	4 months (24 weeks)	Measuring the Little’s irregularity index (mm) in the lower arch, before bonding and at each archwire changing visit, which was made every 6 weeks until the end of the alignment stage directly in patients’ mouth using a four-digit caliper	Mean mesial tooth displacement was CG:1.3 ± 0.544, EG1: 1 ± 0.28, EG2: 0.9 ± 0.155, EG3:1.25 ± 0.866. No statistically significant difference (*p* < 0.05) was detected between the experimental groups at any time point	OTM is not influenced by etoricoxib, acetaminophen, or ibuprofen when prescribed with their recommended doses for three days after each archwire placement
14. Hammad et al. [29], 2012	Rats, 40 randomly divided into 4 groups of 10 each	Celecoxib Ketorolac Paracetamol	50 g from a precalibrated closed Sentalloy coil spring between the upper left first molar and the two upper incisors	Gastric gavage, once a day for 2 consecutive months CG: reverse osmosis water EG1: 10 mg/kg celecoxib EG2: 3 mg/kg ketorolac EG3: 150 mg/kg paracetamol	2 months	Measuring the relative separation between M1 and M2 (mm) intraorally using Vernier calipers before appliance insertion and immediately after sacrifice Effect on bone resorption using immunohistochemical staining of MMP-13	Mesial tooth displacement was CG: 1.78 ± 0.43, EG1: 1.81 ± 0.43, EG2: 1.136 ± 0.28, EG3: 1.08 ± 0.27. The differences were statistically significant (*p* < 0.001). The mean number of MMP-13-positive osteoclasts was highest in EG1, followed by CG, and was decreased in EG2 and EG3	OTM and bone resorption were not influenced by celecoxib administration
15. Stabile et al. [54], 2009	Wistar rats, 30, distributed in 2 groups of 15 each	Acetaminophen Celecoxib	Activated orthodontic appliance on the upper incisors (30 g on each tooth) that was left for 48 h (appliance group) or was immediately removed after insertion (control group)	Oral gavage with 1 mL solution of drug 30 min before and 12, 24, and 36 h after fixation of the appliance CG: without orthodontic appliance + carboxymethylcellulose EG1 (CEL): celecoxib 50 mg/kg EG2 (ACET): acetaminophen 200 mg/kg EG3 (CMC): carboxymethylcellulose 0.4%	2 days	Quantification of the inter-incisal gap (mm) by digitalized photographies of the maxilla using the Image J program	In EG1 (1.11 ± 0.05) and EG2 (1.22 ± 0.04), the inter-incisal gap was not affected (*p* > 0.05) as compared to the control groups	OTM was not affected by celecoxib use for 2 days
16. Jerome et al. [53], 2005	Wistar rats, 20, divided into 3 groups	Celecoxib	80 g from a nickel–titanium closed-coil spring with an additional spring eyelet between the first molar and incisors	Oral administration of drinking water CG: no treatment EG1: 25 mg/kg celecoxib CG2: 50 mg/kg celecoxib	2 weeks	OTM was measured as the distance between M1 and M2	No differences were found between the three groups of rats (0.5 mm/two weeks)	OTM was not influenced by celecoxib administration
17. Gameiro et al. [55], 2008	Wistar rats, 32, divided into 4 groups: Groups I and II—9 rats each; Groups III and IV—7 rats each	Celecoxib	50 g from a closed-coil nickel–titanium spring between the maxillary first molar and incisors	Intraperitoneal injections, 2 h before appliance placement, and postoperative doses for 2 days CG1 (Group I): saline injections on days 1, 2, and 3 EG1 (Group II): celecoxib (10 mg/kg) twice a day on days 1, 2, and 3 CG2 (Group III): saline injections on days 1 to 14 EG2 (Group IV): celecoxib (10 mg/kg) on days 1 to 14	2 weeks	The distance between the mesial surface of M1 and the distal surface of M3 was measured bilaterally with an electronic caliper under a dental operating microscope The osteoclasts were counted at the alveolar bone surface (compression side) adjacent to the entire mesial root by histochemistry	OTM was significantly reduced in EG1 and EG2 compared to CG (*p* = 0.0009). The difference between times of treatment was also significant (*p* = 0.0430). The number of osteoclasts did not differ between drugs or times of treatment (*p* = 0.1230; *p* = 0.4014)	OTM was reduced by both short- and long-term celecoxib administration
18. Sodagar et al. [56], 2013	Rats, 28, divided into 4 groups of 7 each	Celecoxib	60 g from a closed nickel–titanium coil spring between the right maxillary first molar and incisors, activated only once at the beginning of the study	Local subperiosteal injections in the buccal mucosa of the upper right M1 at 72 h intervals starting from the first day of appliance insertion to the 18th day (3 days before the end of the study) CG1 (Group 1): no injections EG1 (Group 2): celecoxib (0.3 mg in 0.1 mL saline solution) CG2 (Group 3): normal saline injections (0.1 mL saline solution) CG3 (Group 4): needle penetration into the subperiosteal space without injecting any solution	3 weeks	Measuring the space (mm) between the right M1 and M2 with a standard interproximal feeler gauge before appliance removal to avoid any probability relapse Histological study to evaluate root resorption	OTM in EG1 (0.21 ± 0.06) was significantly lower than CG1, CG2, and CG3 (0.54 ± 0.08, 0.51 ± 0.04, 0.58 ± 0.06, respectively). The mean osteoclast counts significantly decreased in EG1 when compared with the other groups	OTM and the number of osteoclasts decreased after celecoxib administration
19. Gonzales et al. [39], 2009	Wistar rats, 60, randomly divided into 12 groups of 5 each	Aspirin Acetaminophen Meloxicam Celecoxib Prednisolone	50 g from a NiTi closed-coil spring between the maxillary left molar and the incisors	Oral administration (via drinking water) CG1 (negative control): neither pharmacologic treatment/tooth movement CG2 (positive control): no pharmacologic treatment, but orthodontic treatment EG1: aspirin (high dose 300 mg/kg) EG2: aspirin low dose (60 mg/kg); EG3: acetaminophen (high dose 100 mg/kg) EG4: acetaminophen (low dose 20 mg/kg) EG5: meloxicam (high dose 67 mg/kg) EG6: meloxicam (low dose 13 mg/kg) EG7: celecoxib (high dose 16 mg/kg) EG8: celecoxib (low dose 3.2 mg/kg) EG9: prednisolone (high dose 0.67 mg/kg) EG10: prednisolone (low dose 0.13 mg/kg)	2 weeks	The change in the distance (mm) between the most posterior point of the posterior border of the maxillary first molar crown and the most anterior point of the anterior border of the maxillary second molar crown on digitized lateral cephalometric radiographs	Mean mesial tooth displacement was CG1: 0 CG2: 0.28 EG1: 0.24 EG2: 0.28 EG3: 0.25 EG4: 0.27 EG5: 0.25 EG6: 0.26 EG7: 0.16 EG8: 0.20 EG9: 0.07 EG10: 0.15	Administration of high and low doses of celecoxib reduces OTM in rats, while aspirin, acetaminophen, and meloxicam do not seem to affect OTM
20. Sari et al. [31], 2004	Humans, 36, divided into 3 groups of 12 each	Aspirin Rofecoxib	120 g from a nickel–titanium closed-coil spring between the maxillary canines and second premolars	Oral administration CG: no intervention EG1: aspirin 500 mg, 3 times daily, for 2 days EG2: rofecoxib 25 mg on the day of archwire activation and 12.5 mg on the next day	7 days	Evaluating the PGE2 levels (pg/L) in the GCF measured with automated enzyme immunoassay GCF was sampled after the activation of the coil spring (T0) and at 24 (T1), 48 (T2), and 168 h (T3)	No statistically significant difference was observed between the rofecoxib and control groups at any time point. PGE2 levels at T1 were CG: 75.8, EG1: 64.7, EG2: 74.2	OTM was not inhibited by rofecoxib administration on the first day of the experiment
21. De Carlos et al. [47], 2006	Wistar rats, 42, divided into 6 groups of 7 each	Diclofenac sodium Rofecoxib	50 or 100 g from a unilateral closed-coil spring, stretched between the maxillary left first molar and the incisor	injections in the maxillary gingiva, close to the first molar CG1 (CG-50): 50 g force and 0.9% saline solution injections EG1 (R-50): 50 g force and 2 injections of 1 mg/kg bw of rofecoxib on day 1 and day 3 EG2 (D-50): 50 g force, 10 mg/kg bw diclofenac CG2 (CG-100): 100 g force and same saline solution injection EG3 (R-100): 100 g force and same rofecoxib treatment EG4 (D-100): 100 g force and same diclofenac treatment	10 days	The distance between the first and second molar (mm) on lateral cranial teleradiographic images	Mesial tooth displacement was CG1: 0.43 ± 0.13, CG2: 0.72 ± 0.14, EG3: 0.19 ± 0.13 No movement was found in EG1, EG2, and EG4	Using selective COX-2 inhibitors rather than nonspecific COX inhibitors to avoid interference with OTM seems to have no advantage since both have an inhibitory effect on OTM
22. De Carlos et al. [52], 2007	Wistar rats, 28, divided into 4 groups CG—12 rats EG1—5 rats EG2—6 rats EG3—5 rats	Rofecoxib Celecoxib Parecoxib	50 g from a unilateral closed-coil spring, stretched between the maxillary left first molar and the incisor	3 injections in the maxillary gingiva, close to the first molar, on the day of appliance placement, on day 3 and day 5 by dissolving tablets in saline solution CG: equivolumetric 0.9 per cent saline solution EG1: 0.5 mg/kg bw of rofecoxib EG2: 8 mg/kg bw celecoxib EG3: 25 mg/kg bw parecoxib	10 days	The distance between the first and second molar (mm) on lateral cranial teleradiographic images	Mesial tooth displacement was CG: 0.33 ± 0.07, EG2: 0.42 ± 0.09, EG3: 0.22 ± 0.04 No movement was found in EG1	Celecoxib and parecoxib, but not rofecoxib, are appropriate for discomfort and pain relief while avoiding interference during OTM

bw—bodyweight, CBCT—cone beam computed tomography, COX—cyclooxygenase, CG—control group, CT—computed tomography, EG—experimental group, ELISA—enzyme-linked immunosorbent assay, g—grams, GCF—gingival crevicular fluid, h—hour, kg—kilograms, M—molar, mg—milligrams, mm—millimeters, ml—milliliters, µg—micrograms, MMP—metalloproteinase, OPG—osteoprotegerin, OTM—orthodontic tooth movement, pg/L—picogram/liter, PGE2—prostaglandin 2, RANK–L—receptor activator of nuclear factor kappa-Β ligand, T—time.
